# Influence of Metal Concentration and Plumbing Materials on *Legionella* Contamination

**DOI:** 10.3390/microorganisms10051051

**Published:** 2022-05-19

**Authors:** Anita Rakić, Darija Vukić Lušić, Anamarija Jurčev Savičević

**Affiliations:** 1 Public Health Institute of Split-Dalmatia County, Vukovarska 46, 21000 Split, Croatia; anita.rakic@nzjz-split.hr; 2 Department of Environmental Health, Faculty of Medicine, University of Rijeka, Braće Branchetta 20, 51000 Rijeka, Croatia; 3 Department of Environmental Health, Teaching Institute of Public Health of Primorje-Gorski Kotar County, Krešimirova 52a, 51000 Rijeka, Croatia; 4 Center for Advanced Computing and Modeling, University of Rijeka, Radmile Matejčić 2, 51000 Rijeka, Croatia; 5 Department of Health Studies, University of Split, 35 P.P. 464, Ruđera Boškovića Street, 21000 Split, Croatia

**Keywords:** *Legionella*, Legionnaires’ disease, biofilm, preventive measures, hot water, water distribution system

## Abstract

*Legionella* colonization of water supply pipes is a significant public health problem. The objective of this work was to evaluate *Legionella* colonization in hotel hot water systems and to investigate the relationship between metal concentrations, piping materials (galvanized iron pipes and plastic pipes), and *Legionella* proliferation. Concentrations of calcium and magnesium ions and the presence of *Legionella pneumophila* were determined in a total of 108 water samples from the hot water systems of four hotels in Split-Dalmatia County over a 12-month period, and additional data on piping materials were collected. *L. pneumophila* was isolated in 23.1% of all samples—in 28.8% (15/52) of water samples from galvanized iron pipes and in 17.8% (10/56) of samples from plastic pipes. *L. pneumophila* serogroups 2–14 were isolated from all samples. This study found higher prevalence of *L. pneumophila* at higher concentrations of Ca and Mg ions (except for Mg and plastic pipes). The metal parts of the water supply may be important factors in *Legionella* contamination due to the possibility of lime scale or roughness of the pipes. Higher Ca and Mg ion concentrations increased the risk of *Legionella* colonization.

## 1. Introduction

The health problems associated with *Legionella* have gained increasing public and professional attention. Legionellosis is a series of infections, ranging from mild febrile illness (Pontiac fever) to severe pneumonia with potentially fatal outcomes (Legionnaires’ disease), caused by *Legionella pneumophila* and related *Legionella* bacteria and often associated with travel [1]. *L. pneumophila* is transmitted by inhalation of contaminated aerosols produced by a variety of devices such as water systems in various accommodation sites, homes, ships, and buildings; cooling towers; spa pools; fountains; misting devices; and others.

*Legionella* is ubiquitous in natural and artificial water environments. A good understanding of the factors that influence *Legionella* survival and growth is important in controlling the bacteria in artificial water systems. The technical characteristics of water distribution systems (WDS), such as the types of pipe materials, pipe diameters, flow patterns, and chemical and microbiological processes, can cause a variety of water quality problems and increase the risk of various infections, including *Legionella*. Microorganisms in a WDS attach to the surface/walls of the water system by forming biofilms, and their formation is influenced by microflora, sediment, hydrodynamic conditions, and temperature within the system, among other factors [2,3,4]. Biofilm is actually an ecological niche that provides microorganisms with structure, stability, nutrients, and protection from turbulent flow and disinfectants [5,6]. Therefore, microorganisms in biofilms often exhibit increased tolerance to biocides (up to 1000 times) [7] and are difficult to remove, especially from unavailable surfaces (blind ends). The concentration of free residual chlorine required to inactivate *Legionella* is 0.2 mg/L, while a minimum of 1–2 mg/L is required to kill them [8].

Scaling occurs in water supply pipes in WDSs, especially on the surfaces of the heat transfer system, and this is particularly evident in pipes distributing water with increased hardness [9,10]. Water scale is present in all parts of water supply systems, and the porous structure of water scale favors the proliferation of opportunistic pathogens (e.g., *Legionella* spp.) and protects against the effects of disinfectants [11]. The consequences of biofilm and lime formation in large systems are weaker water flow and difficulties in maintaining prescribed temperature levels for both hot and cold water, conditions which favor the growth of *Legionella* [12].

Physical, chemical, and biological indicators of water quality are interrelated. In general, corrosion and sediment retention primarily occur in metallic pipelines, increasing the concentration of nutrients which are needed for biofilm formation and the surface roughness of the inner pipe [13]. In 2011, Bargellini et al. studied the colonization rate of *Legionella* spp. as a function of the type of material from which water pipes were made. Their results showed lower concentrations of microorganisms on smooth pipes than on rough surfaces.

The aim of this study is to evaluate whether environmental factors (calcium and magnesium concentration) and different types of construction materials of hot water distribution pipes influence the occurrence of *L. pneumophila* in WDSs. The results may be useful for efforts to improve preventive measures and minimize the risk of *Legionella* infection.

## 2. Materials and Methods

A total of 108 water samples were collected over a 12-month period from the hot water system of four hotels in Split–Dalmatia County. All hotels are supplied with water from the public water supply system connected to underground aquifers. The spring water is of the Ca-HCO_3_ type with low mineralization, yields relatively little residue on evaporation, and has a moderate total hardness of 11.9 to 12.3 German degrees (°D) and a carbonate hardness of 10.3 to 11.0 °D. The average water temperature is 12.3 °C, and the pH is 7.5. In the dry part of the year, it contains slightly more chloride than typical karst waters. The average annual concentration of chloride in the water is 13–22 mg/L, and the average annual concentration of sulfate in the water is 8–36 mg/L. It contains a low concentration of dissolved carbon dioxide (about 5 mg/L). The dissolved oxygen concentration and biochemical oxygen demand (BOD5) in the spring water meet drinking water standards (about 1.03 mg O_2_/L). The spring water is turbid above the allowable level (4 NTU) on an average of 8–13 days per year. Greater turbidity of the water occurs during and immediately after heavy rains and usually passes quickly. The water is purified by precipitation, filtration, and disinfection with chlorine gas.

### 2.1. Sampling of Water for Analysis

Chlorinated water samples for chemical and microbiological analysis were collected in sterile bottles with Teflon caps (volume of 1000 mL), with mandatory flaming of the WDS taps. Samples were placed in bottles containing 0.1 mL of a 10% sodium thiosulfate solution (Na_2_S_2_O_3_). Post-flush samples were placed in bottles sealed with stoppers (EN ISO 19458:2006) [14]. The water samples were delivered to the laboratory for analysis within six hours. Before delivery, they were stored in a manual refrigerator at +4 °C.

### 2.2. Chemical and Microbiological Analysis

Water temperature was measured in accordance with the APHA Standard Methods for the Examination of Water and Wastewater (22nd Edition, 2012), 2550 B (American Public Health Association—APHA, American Water Works Association—AWWA, Water Environment Federation—WEF). Residual free chlorine was measured according to EN ISO 7393-2:2000 (Water quality—Determination of free chlorine and total chlorine—Part 2: Colorimetric method using N,N-diethyl-1,4-phenylenediamine), and pH was measured in accordance with EN ISO 10523:2012 (Water quality—Determination of pH). Prior to chemical analysis (determination of Ca and Mg ions) samples were acidified with 65% HNO_3_. The mass concentrations of these ions were determined on an atomic absorption spectrophotometer (AAS Z–2000, Hitachi, Japan).

The microbiological analysis of the samples was carried out in the microbiological reference laboratory immediately after the delivery of the samples and within a maximum of 24 h after sampling. Cultivation and identification of *Legionella* was performed according to ISO 11731-2:2004 [15].

Bacterial concentration analysis was performed by membrane filtration of 100 mL of the sample using a 0.20 µm pore size membrane (90 mm diameter, a polyamide filter, Millipore, Bedford, MA, USA). The filter was treated with an acidic buffer (30 ± 5 mL) for 5 min, rinsed with Page’s saline (20 ± 5 mL), and transferred to a selective medium, BCYE agar (Buffered Charcoal Yeast Extract agar bioMériux, Marcy l’Etoile, France) for up to 10 days at 36 °C. *Legionella* spp. Colonies grown on BCYE agar were of different colors (white, purple, blue, or lemon green), luminescent, and round-shaped with continuous edges. Morphologically typical *Legionella* spp. Colonies were subcultured on BCYE agar and BCYE agar without L-cysteine (BCYE-Cys) for >2 days at 36 °C. Because *Legionella* are known to grow in the presence of cysteine, colonies with characteristic morphological features that grew on BCYE agar but not on BCYE-Cys medium were considered as presumptive *Legionella*. Colonies grown on BCYE agar were confirmed with an agglutination test (*Legionella* latex test, Oxoid, Basingstoke, UK), which allows differentiation of serogroups of *L. pneumophila* (1 and 2–14). If there was only one colony type, five presumptive colonies of *Legionella* were tested by agglutination. If there were more morphologically different types of presumptive *Legionella* colonies, at least three colonies of each type were confirmed by agglutination. The results were expressed in CFU/L, and the detection limit of the method was 10 CFU/L.

### 2.3. Additional Information on the Water Supply Network

Additional information on the type of the internal water supply network was collected through surveys.

### 2.4. Statistical Analysis

The results are presented using descriptive statistics, relative frequency, arithmetic mean and median as measures of mean, standard deviation (SD), and interquartile range (IQR) as a measure of dispersion of data. Statistical analyses were performed using the MedCalc package version 11.3.0.0; Windows 2000/XP/Vista/7 versions (SPSS Inc., Chicago, IL, USA) and the TIBCO Statistica 13.5.0 software package (TIBCO Software Inc., Palo Alto, CA, USA). Prior to the statistical analysis, normality tests were performed to check the data distribution. Nonparametric tests (Spearman’s correlation coefficient, the Mann–Whitney U test, and the Kruskal–Wallis H test) were used because the data did not follow a Gaussian distribution. The Spearman correlation coefficient (ρ) was used to determine the relationship between metal concentrations and the presence of *Legionella* spp. The chi-square (χ^2^) test was used to determine statistically significant differences between *Legionella* contamination and the type of construction material used in the internal networks of the WDS. Statistical results were interpreted at the significance level *p* < 0.05.

## 3. Results and Discussion

This study investigated the colonization of hotel water systems with *Legionella* as a function of plumbing materials and metal concentration in four hotels in Split–Dalmatia County. A total of 108 hot water samples were analyzed for chemical (Ca and Mg ion concentration) and microbiological (*Legionella*) water quality parameters. Regarding the material of the plumbing systems, two accommodation sites had plastic pipes (PVC) (56 hot water samples), while the other half had galvanized pipes (52 hot water samples). All colonies that were suspected of being *Legionella* were confirmed as *L. pneumophila* of serogroups 2–14. Increased resistance of *L. pneumophila* SGs 2–14 to SG 1 and *Legionella* spp. to environmental conditions has also been confirmed in studies in Italian hospitals and prisons [16,17]. *L. pneumophila* was isolated in 23.1% (25/108) of the total samples, with a range from 50 CFU/L to 3000 CFU/L. Previous studies reported widely varying proportions of *Legionella*-positive samples in hotel and public building hot water systems: 3.1% in a study in Japan [18], 8.5% in Spain [19], 18.5% in Greece [20], 51.7 in Morocco [21], and 60.5% in Italy [22]. Table 1 shows that 15 (28.8%) water samples from galvanized iron pipes were positive for *L. pneumophila* compared with 10 (17.8%) positive samples from plastic pipes, which was not statistically significant.

**Figure 1:**
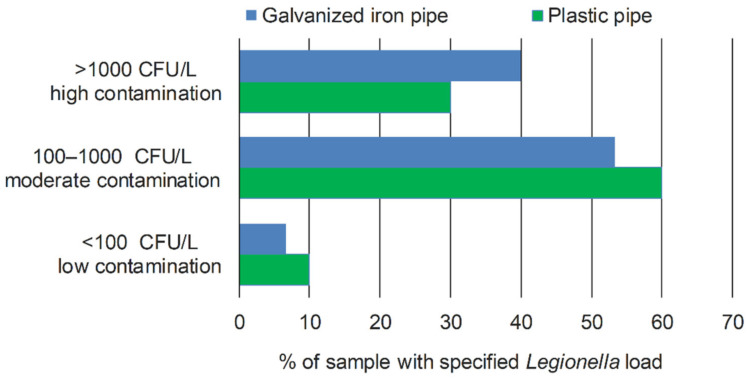
Percentage of *Legionella*-positive water samples in relation to the *Legionella* load for galvanized pipe and plastic (PVC) pipe.

However, samples from galvanized iron pipes had a statistically significantly higher load of *L. pneumophila* (χ^2^ = 4.502; *p* = 0.034). Figure 1 shows that a higher proportion of galvanized pipe samples fell into the highest contamination category.

**Figure 2:**
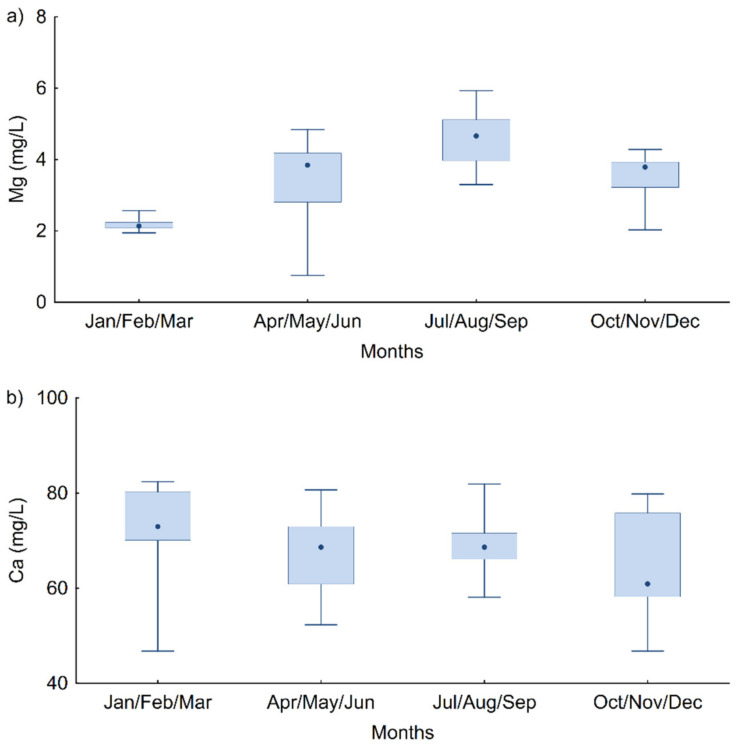
Median values and ranges of concentrations of (**a**) Mg and (**b**) Ca ion, in all water samples in each quarter of the year.

The highest percentage of positive samples for *L. pneumophila* (40.4%) was detected in the warmer season, from July to September, and the lowest (19.7%) in the 2nd quarter (April–June), compared to the other quarters of the year. In addition, the results of this study indicate seasonal variability in magnesium concentration in the WDS, which was statistically significant and highest in the 3rd quarter (K–W test, H(3, *n* = 108) = 47.917, *p* < 0.0001) (Figure 2a). This can be explained by the fact that due to the continuously increased water consumption in the summer months, there is a permanent release of already formed deposits or water scale pieces from the pipe walls of the WDS, which manifests as a higher concentration of metal ions in hot water samples. Hence, the period of the highest number of positive samples for *L. pneumophila* coincides with the period of the highest Mg concentration. These results support recent research indicating that the seasonal variability of environmental influence is very interesting and variable [23,24]. Pinto and Raskin suggested in their 2012 study [25] that the most influential variables in water chemistry may change with the seasons. In contrast to Mg ion concentrations, Ca ion concentrations were statistically significantly higher in the 1st quartile (January–March; K–W test, H(3, *n* = 108) = 13.765, *p* = 0.0032) than in the 2nd and 4th quartiles (Figure 2b).

**Figure 3:**
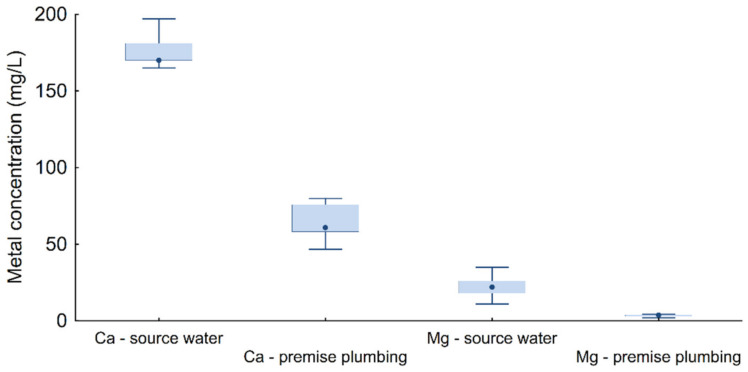
Metal concentration (Ca and Mg) in Jadro source water and premise plumbing.

The concentrations of calcium and magnesium in the water source Jadro were statistically significantly higher (173.3 mg/L, *n* = 44 and 25.1 mg/L, *n* = 108, respectively) than in the plumbing premises (25.1 mg/L, *n* = 44 and 3.5 mg/L, *n* = 108, respectively), according to the Mann–Whitney U test, *p* < 0.001 (Figure 3). Accordingly, there were decreases of Ca and Mg ions in the plumbing system by 85% and 86%, respectively, compared to the concentrations of those metals in the Jadro water source. There were no seasonal changes in the concentrations of the observed metals in the spring water, in contrast to the concentrations of metals in plumbing premises, where seasonal variations were observed. The concentration of Ca and Mg decreased due to the formation of pipe scale at higher hot water temperatures (>50 °C), which are targeted in the water supply system of building owners to reduce the risk of *Legionella* and other opportunistic pathogens. Scale is a precipitation of CaCO_3_ in drinking water systems, which leads to clogging of the pipes and reduces the efficiency of heat transfer. PVC pipes generally have lower scale potential than metal pipes [26]. Surprisingly, chlorine concentration values in the warmest part of the year (3rd quarter) were significantly higher than in the 1st and 4th quarters, considering that higher water temperature accelerates the rate of chlorine degradation in water distribution systems [27].

**Figure 4:**
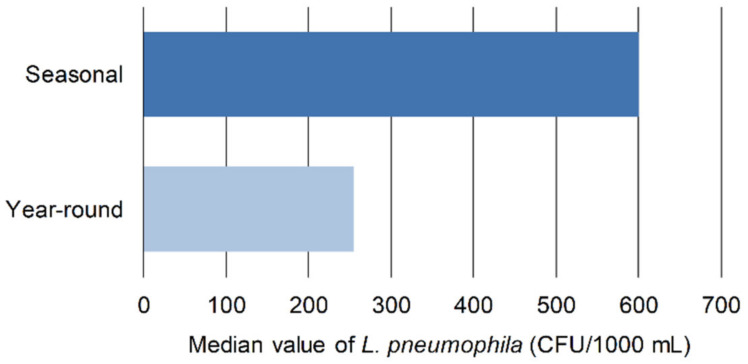
Median of *Legionella* concentration (CFU/1000 mL) by year-round vs. seasonal facilities.

The results also show that the WDS is more contaminated with *Legionella* in hotels that are open seasonally than in hotels that are open year-round (Figure 4) as well as in older hotels.

Table 2 shows the values of the analyzed parameters and characteristics of all four hotels studied. In addition, the concentration of *Legionella* was statistically significantly highest (K–W test, H(2, *n* = 108) = 23.999, *p* < 0.0001) in systems with a hot water temperature below 50 °C (mean ± SD, 382.9 ± 707.6 CFU/100 mL), followed by samples with a temperature in the range of 50–60 °C (mean 100.0 ± 307.5 CFU/100 mL), while no *Legionella* were detected in samples with a temperature above 60 °C. Of the 25 *L. pneumophila*-positive samples, only 5 had chlorine concentrations greater than 0.2 mg/L, with pH values significantly higher (median 8.3, IQR 8.1–8.4) than those of the negative samples (median 8.1, IQR 8.0–8.3) (M–W test, Z = −2.222, *p* = 0.026). Correlation analysis showed a significant negative correlation between *L. pneumophila* and water temperature (ρ = −0.529, *p* < 0.05) and a significant positive correlation between pH and magnesium ion concentration (ρ = 0.208, ρ = 0.2016, *p* < 0.05, respectively).

The Ca and Mg concentrations of the hot water samples ranged from 46.78–82.4 mg/L and 0.75–5.93 mg/L, respectively (Figure 2). The largest interquartile ranges of the calcium and magnesium concentrations were observed in the 4th quarter (October–December) and 2nd quarter (April–June) samples, respectively. The smallest interquartile ranges for Ca and Mg were found in the 3rd quarter (July–September) and 2nd quarter (April–June), respectively. The results of this study show that in water samples in which *L. pneumophila* were detected, Ca ion concentrations were statistically significantly higher than in negative samples (Mann–Whitney U test, *p* = 0.027), while Mg ion concentrations were also higher, but the difference was not statistically significant. These results are consistent with previous studies [18,22].

The presence of different metal ions in water and the influence of their concentrations on *Legionella* spp. colonization have been investigated in numerous studies, and the results suggest that certain ions prevent *Legionella* spp. growth and development, while others have a biostimulatory effect [18,28,29]. Ca and Mg ions are involved in biofilm formation by enhancing adhesion of *Legionella* to the surface [30,31]. Studies in Morocco [32] revealed that *L. pneumophila* SGs 2–15 have a higher capacity to colonize plumbing materials used mainly in domestic water systems compared to *L. pneumophila* SG 1. Ji et al. [33] considered Mg concentration as an indicator of salinity, while several studies found salinity to be the strongest determinant of microbial community [34,35]. Although it is known that magnesium ions are essential nutrients for the growth of *Legionella* [36], the influence of Mg in water is still a topic of scientific interest. Some studies [37,38] found no correlation between calcium and magnesium concentrations and the presence of *Legionella* spp. Vickers et al. [39] found a positive correlation, while Leoni et al. [28] found a statistically significant inverse correlation between Ca and Mg ion concentrations and the presence of *Legionella* spp. and *L. pneumophila*.

What we know so far is that plumbing materials affect biofilms in terms of bacterial diversity [40] or formation rate, which could indirectly affect the composition of the bacterial communities in water. The results of this study show that Mg ion concentrations were statistically higher in galvanized pipes than in PVC pipes (K–W test, H(3, *n* = 108) = 32.688, *p* < 0.0001). There were no differences in Ca ion concentrations or in the presence of *L. pneumophila* when differences in piping materials were considered.

**Figure 5:**
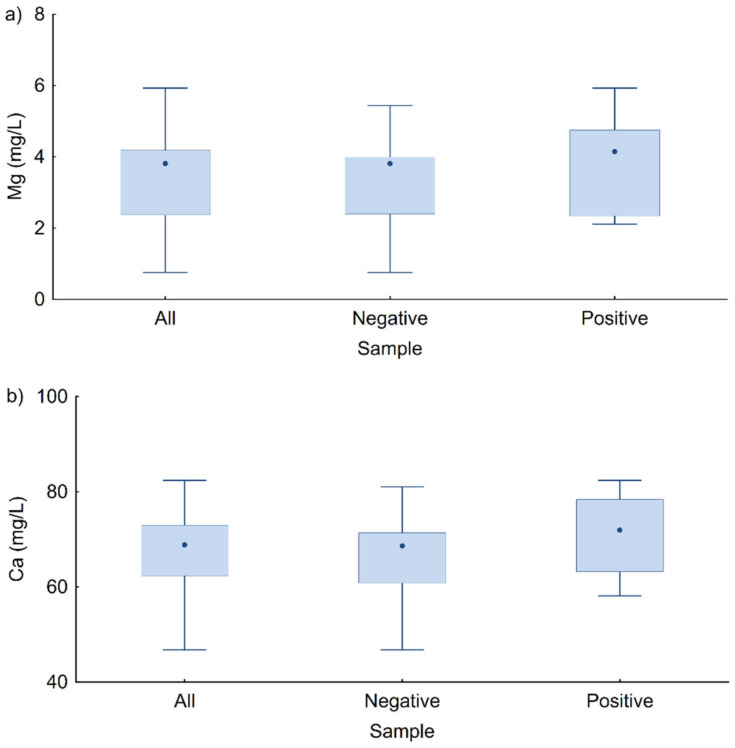
Interquartile representation of concentration of (**a**) Mg ion and (**b**) Ca ion, for all hot water samples and for samples with negative and positive findings of *L. pneumophila*.

The concentrations of Mg and Ca ions in samples with/without *L. pneumophila* are shown in Figure 5. The median Mg ion concentration was higher in the positive samples, but the difference was not statistically significant (Figure 5a), while there were statistically significant differences between the concentrations of Ca ions in the *L. pneumophila*-positive and negative samples (M–W test, Z = −2.207, *p* = 0.027; Figure 5b). Higher interquartile ranges of calcium and magnesium concentrations and higher values of their medians were observed in the samples with isolated *L. pneumophila* compared to the *L. pneumophila*-negative samples. It is also evident that the median values of Ca and Mg concentrations in the positive samples were in the middle of the interquartile range or just below the upper quartile, while in the negative samples, the median values for calcium and magnesium were closer to the upper value of the interquartile range.

**Figure 6:**
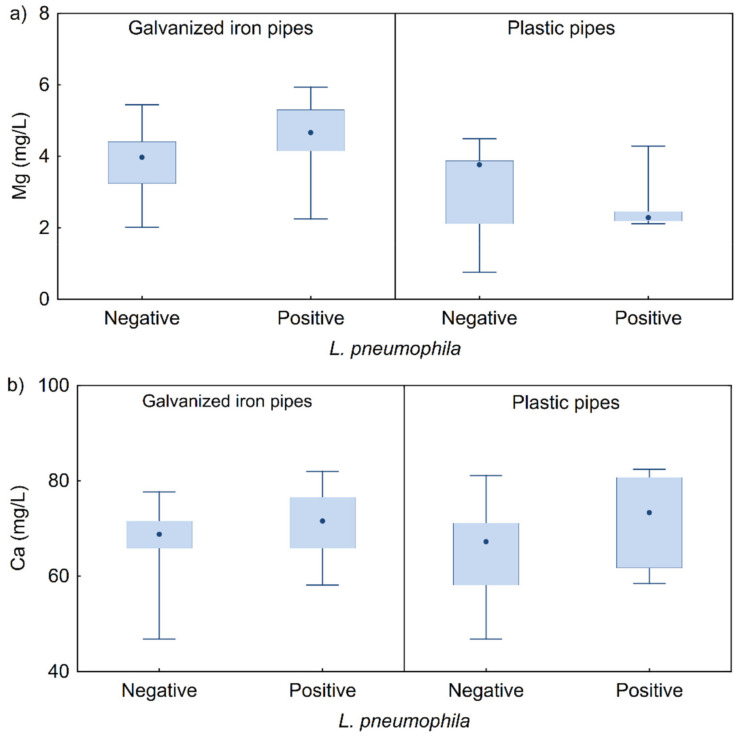
Negative and positive *L. pneumophila* samples as a function of concentration of (**a**) Mg and (**b**) Ca ion (interquartile range—IQR), in water from the galvanized and plastic piping systems.

Statistical analysis showed significantly higher concentrations of Mg ions in water samples positive for *L. pneumophila* from galvanized iron pipes compared to positive and negative samples from plastic pipes (K–W test, H(3, *n* = 108) = 32.687, *p* < 0.0001), with no difference compared to negative samples from galvanized pipes (Figure 6a). The median values of Ca ion concentrations in both types of pipes were higher in samples with isolated *L. pneumophila* but with no statistically significant difference, with interquartile ranges of results being higher in PVC pipes (Figure 6b).

During the study period, no cases of Legionnaires’ disease were associated with the hotels studied. One of the possible factors influencing this result is the dominance of metal components in the external distribution network. The same metal components are installed in the main distribution network of the WDS, namely water supply elements, pumping stations, water treatment plants, and water supply systems (pipelines with associated fittings and water supply armature). It can be concluded that the type of internal water supply networks has little influence on the metal ion concentration with respect to the main distribution network of the WDS. This is consistent with previous studies [22,41] that have demonstrated the relationship between corrosion products from metal segments of water supply systems and the formation of biofilms in those segments. In addition, biofouling leads to an undesirable accumulation of microbiological deposits at the interface and creates optimal conditions for the survival and growth of *Legionella* spp. [11]. Although there was no statistically significant difference in the proportion of *Legionella*-positive samples between the water system of galvanized pipes and that of plastic pipes, it is evident that galvanized pipes have a higher concentration of metal ions in the presence of *L. pneumophila* compared to plastic pipes.

It can be concluded that galvanized systems pose a greater risk for biofilm development and *L. pneumophila* survival compared to plastic hot water distribution systems. According to studies by Stout and co-workers [38], several processes occur on the surface of a galvanized material in contact with a water medium: corrosion (including microbial-induced corrosion), the formation of scale leading to the degradation of the metal, and the formation of biofilms. This leads to an undesirable accumulation of microbiological species on the surface of water pipes. These results are confirmed by other studies [7,13,22,42], which showed that the construction material of the drinking water distribution pipe affects the development of microorganisms. The porous structure of lime also favors the proliferation of microorganisms by protecting them from the influence of disinfectants and the influence of hot water [43].

To minimize the risk of Legionnaires’ disease, it is necessary to reduce the factors that affect the survival and growth of *Legionella* in drinking water installations, including metal ion concentrations. Preventive measures depend on the chemical composition of the water as well as on the properties of the material from which an external and/or internal distribution network for the WDS is made. It is important to keep all parts of the WDS safe, especially in terms of construction materials and maintaining working conditions. Low water flow, water stagnation, temperature, and corrosion of metal pipes and fittings are some of the factors that favor the growth of *Legionella*, especially in the hot water supply.

## 4. Conclusions

A higher proportion of positive *L. pneumophila* samples were found in galvanized pipes compared to PVC pipes, although the difference was not statistically significant. However, samples from galvanized iron pipes show statistically significantly higher *L. pneumophila* contamination. The concentration of Mg ions is statistically significantly higher in samples from galvanized pipes than in samples from PVC pipes, while the Ca ion concentration was higher in samples from PVC pipes, but not statistically significantly so. In addition, seasonal trends of *L. pneumophila* were observed in water distribution systems, and the highest percentage of positive samples was found in the warmest quarter of the year, which coincided with the highest concentrations of magnesium ions. The prevalence of *L. pneumophila* in the hot water distribution system was found mainly in seasonally open, older hotels with temperatures below 50 °C and higher pH (chlorine concentration below 0.2 mg/L). The metal parts of the water supply may be important factors in *Legionella* contamination due to the possibility of scale deposition or roughness of the pipes. The data indicate a large loss of the studied metals in the source water (up to 85%) compared to plumbing premises, which is related to the formation of scale in the distribution network. This study shows that monitoring and determining risk factors for the presence of *L. pneumophila* is important to minimize the risk of Legionnaires’ disease. The results obtained can be used to inform decisions on protective measures to reduce and eliminate the presence of *L. pneumophila*.

## Figures and Tables

**Table 1:** Display of negative and positive findings of *Legionella* spp. in water samples collected from hotels with different type of pipes.

Presence of *L. pneumophila*	Number of Samples	Types of Pipes	
		Galvanized Iron	Plastic
Negative samples	N	37	46
	%	71.2	82.2
Positive samples	N	15	10
	%	28.8	17.8
Total number of samples		52	56

**Table 2:** Analyzed water parameters and characteristics of four hotels studied.

Facility	T (°C) *	pH	Cl_2_ (mg/L)	Ca (mg/L)	Mg (mg/L)	Opening Regime	Maintenance Regime
**Hotel 1**	51.0 ± 8.4	8.07 ± 0.30	0.16 ± 0.08	66.0 ± 10.4	3.2 ± 0.9	year-round	(1) keeping cold water temperature <20 °C and hot water temperature >50 °C; (2) before the start of the new season the system was pasteurized and hyperchlorinated; (3) weekly flushing of outlets for at least 5 min
**Hotel 2**	53.0 ± 5.0	8.24 ± 0.15	0.21 ± 0.02	67.7 ± 6.6	4.3 ± 0.8	seasonally	
**Hotel 3**	54.6 ± 4.8	8.12 ± 0.12	0.22 ± 0.06	67.8 ± 8.1	2.8 ± 1.0	year-round	
**Hotel 4**	53.8 ± 6.0	8.04 ± 0.12	0.23 ± 0.04	69.7 ± 7.2	3.7 ± 4.3	seasonally	

* post-flush sample.

## Data Availability

The data supporting the findings of this study are available within the article.
